# Parental Guidance Suggested: Engaging Parents as Partners in Research Studies of Genomic Screening for a Pediatric Population

**DOI:** 10.3389/fgene.2022.867030

**Published:** 2022-03-25

**Authors:** Sabrina N. Powell, Grace Byfield, Ashley Bennetone, Annabelle M. Frantz, Langston K. Harrison, Erin R. James-Crook, Heather Osborne, Thomas H. Owens, Jonathan L. Shaw, Julianne O’Daniel, Laura V. Milko

**Affiliations:** ^1^ Program for Precision Medicine in Health Care, University of North Carolina at Chapel Hill, Chapel Hill, NC, United States; ^2^ Department of Genetics, University of North Carolina at Chapel Hill, Chapel Hill, NC, United States; ^3^ Community Research Board Member, Durham, NC, United States; ^4^ Community Research Board Member, Mooresville, NC, United States; ^5^ Community Research Board Member, Sanford, NC, United States; ^6^ Community Research Board Member, Chapel Hill, NC, United States

**Keywords:** genomic sequencing, newborn screening, community research board, engaging parents, stakeholders, public health, equity, accessibility

## Abstract

Recent advances in genomic sequencing and genomic medicine are reshaping the landscape of clinical care. As a screening modality, genetic sequencing has the potential to dramatically expand the clinical utility of newborn screening (NBS), though significant barriers remain regarding ethical, legal, and social implications (ELSI) and technical and evidentiary challenges. Stakeholder-informed implementation research is poised to grapple with many of these barriers, and parents are crucial stakeholders in this process. We describe the formation and activities of a Community Research Board (CRB) composed of parents with diverse backgrounds assembled to participate in an ongoing research partnership with genomic and public health researchers at the University of North Carolina. The mission of the CRB is to provide insight into parental perspectives regarding the prospect of adding genomic sequencing to NBS and collaboratively develop strategies to ensure its equitable uptake. We describe how these contributions can improve the accessibility of research and recruitment methods and promote trust and inclusivity within diverse communities to maximize the societal benefit of population genomic screening in healthy children.

## Introduction

Clinical genomic sequencing is increasingly used for diagnosis and management of newborns and children with suspected genetic conditions, but has not been adopted for screening in healthy populations (Biesecker and Green, 2014; Willig et al., 2015; Strande and Berg, 2016). Genomic sequencing has the potential to greatly expand universal newborn screening (NBS) through early diagnosis of rare genetic conditions at birth, thereby enabling early health actions to prevent or ameliorate adverse health outcomes before symptoms develop (Remec et al., 2021). However, substantial ethical, legal, and social implications (ELSI) and practical and policy challenges must be addressed before this technology can be widely adopted for public health screening (Committee on Bioethics et al., 2013; Botkin et al., 2015; Brothers et al., 2019; Ross and Clayton, 2019; Sen et al., 2021). While translational research studies are evaluating various methods of integrating sequencing into NBS (Berg et al., 2017; Holm et al., 2018; Milko et al., 2018; Petrikin et al., 2018; Adhikari et al., 2020; Andrews et al., 2022), effective working partnerships between researchers and community stakeholders are also vitally important to ensure research and future clinical offerings are inclusive, accessible, and beneficial for all (Goldenberg, 2019; Downie et al., 2021; Halley et al., 2022).

Conventional NBS exemplifies the model of public health screening to detect individuals for whom early diagnosis and treatment of “clinically actionable” conditions offers unambiguous health benefits (Berg and Powell, 2015; Hendricks-Sturrup and Lu, 2019; Powell, 2020; Woerner et al., 2021). Expanding NBS via genomic sequencing could dramatically increase the number of clinically actionable conditions that states could effectively screen for, from several dozen to several hundred (Ceyhan-Birsoy et al., 2019; Milko et al., 2019). Rapidly proliferating clinical trials for new gene therapies and pharmaceutical products also promise life-altering interventions for previously untreatable genetic conditions (Tambuyzer et al., 2020). There is growing advocacy for expanding NBS to include genomic sequencing because of the expected impact on health outcomes, and because early initiation of treatment often maximizes health benefits (Kingsmore, 2016; Powell, 2018; Bailey et al., 2021). Public health access to “expanded NBS” could aid efforts to reduce existing disparities in genetic testing and increase equity in potential benefits of a genetic diagnosis, including avoidance of a diagnostic odyssey, access to clinical management and counseling, and reproductive decision-making (Friedman et al., 2017). However, the inherent ambiguity of these benefits, such as enrollment in clinical trials for unproven treatments, and the concomitant potential for harm would likely disrupt the current NBS “opt-out” model and necessitate parental consent (Ross et al., 2013; Botkin et al., 2015).

Studies of stakeholder perspectives about genomic screening indicate that persistent apprehension could impede broad parental consent for expanded NBS, particularly among historically underserved and underrepresented populations (Borry et al., 2008; Shkedi-Rafid et al., 2015; Ulm et al., 2015; Kerruish, 2016; Moultrie et al., 2020; Tutty et al., 2021; Halley et al., 2022). Parental areas of concern include 1) anxiety regarding choices about what information they wish to have disclosed or about the security or potential misuse of their child’s genetic data, 2) the potential for large out-of-pocket expense, 3) future discriminatory implications for their child, and 4) the psychosocial effects of learning about health conditions without affordable or effective treatments (Howard et al., 2015; Paquin et al., 2018). Effectively and equitably integrating genomic sequencing into NBS will require building trust with community partners in diverse settings to understand what genomic information should be returned to parents and how best to communicate that information. Without this crucial insight, limited uptake of genome-scale sequencing is likely and could endanger public trust in the current public health NBS system (Johnston et al., 2018).

Despite these substantial issues and gaps in the clinical evidence base, direct-to-consumer genetic testing has begun targeting healthy infants and children, raising questions about the nature of the information provided to parents (DeCristo et al., 2021). There are currently no standards or guidelines governing disclosure of genomic screening results or follow-up clinical care for those who test positive. Poorly regulated genetic testing poses a significant risk to uninformed parents as well as to primary care providers who will increasingly bear the burden of parental requests for education and information, interpretation of widely variable results, and clinical care among those testing positive for highly heterogeneous conditions (Cohidon et al., 2021; Majumder et al., 2021). Practice-based and stakeholder-informed implementation research is urgently needed to inform and safeguard future public health access to expanded NBS in the face of increasing commercialization.

This article highlights the importance of parent/caregiver engagement in ongoing pediatric genomic screening research and presents a collaborative approach to stakeholder-researcher partnership. As a team, we represent the Community Research Board (CRB), comprising parents from diverse communities in central North Carolina and multidisciplinary genetics professionals (researchers, clinicians, educators, and stakeholder engagement experts) at the University of North Carolina at Chapel Hill (UNC-CH). Together we seek to collaboratively address challenges in designing and broadly implementing research studies of genomic screening and public health offerings for a pediatric population. Here we describe the processes we followed to build a functionally integrated research group of community members and academicians and the activities, and initial outcomes of the CRB. We highlight successes and challenges, as well as key advantages and lessons learned from such a collaboration early in the research process.

## Defining Meaningful Stakeholder Engagement

Stakeholder engagement is a critical component in translational research and includes patients, parents and caregivers, research participants, health care providers, payers, policy makers, advocacy groups and community leaders (Kost et al., 2012; Wilkins et al., 2013; Yarborough et al., 2013; Lemke and Harris-Wai, 2015; Griesemer et al., 2020). Stakeholder engagement in research is defined as the iterative process of actively soliciting the knowledge, experience, judgment, and values of individuals selected to represent a broad range of interests in a particular issue, for the dual purposes of creating a shared understanding and making relevant, transparent, and effective decisions (Deverka et al., 2012). Meaningful engagement empowers stakeholders from the group(s) responsible for or impacted by health and/or healthcare decisions (Concannon et al., 2012) to affect the research process and resulting outcomes (Arnstein, 1969). In this way, stakeholders partner with researchers to collaboratively outline research questions and refine protocols and approaches to address issues that impact their communities.

A well-developed and carefully established bi-directional community research partnership fosters a trusting and mutually beneficial relationship for the research study and the community. In such a collaboration, both researchers and community members are actively involved in the design and implementation of the project as well as the interpretation and dissemination of the findings. Engaged Participation is one category of stakeholder engagement in which community health stakeholders (who traditionally have limited power) collaborate in decision-making and resource allocation with an equitable balance of power that values input from the community health stakeholders (Goodman and Sanders Thompson, 2017). *Transparency, honesty,* and *trust* are key principles of effective engagement when major decisions are made inclusively, information is openly shared, and patients/community members and researchers are committed to open and honest communication (Rawl et al., 2021). The CRB was established following these key principles, with the goal of informing the effective and equitable integration of genomic screening in newborns and children.

## Informing Effective and Equitable Integration of Genomic Screening in Newborns and Children

Recruitment challenges faced by the Newborn Sequencing In Genomic medicine and public HealTh consortium, including the North Carolina Newborn Exome Sequencing for Universal Screening (NC NEXUS) (Roman et al., 2020), NSIGHT1 (Petrikin et al., 2018), and BabySeq (Pereira et al., 2021), suggest substantial stakeholder engagement is necessary to improve enrollment of underrepresented communities in research involving expanded NBS research. Authentic bidirectional involvement with parents from diverse communities is also needed to navigate larger issues and challenges inherent to expanded NBS. Toward this end, we established the CRB as a community-based arm of a research team that also includes investigators and staff from the Program for Precision Medicine in Health Care (PPMH) in the UNC-CH School of Medicine. CRB members were recruited with the expectation that they would be engaged throughout the lifecycle of a research process: 1) developing the research questions, processes, and methods; 2) designing and disseminating informational and educational study materials; 3) participating in community outreach events; and 4) interpreting and disseminating the results from a community perspective.

### Recruitment

**FIGURE 1:**
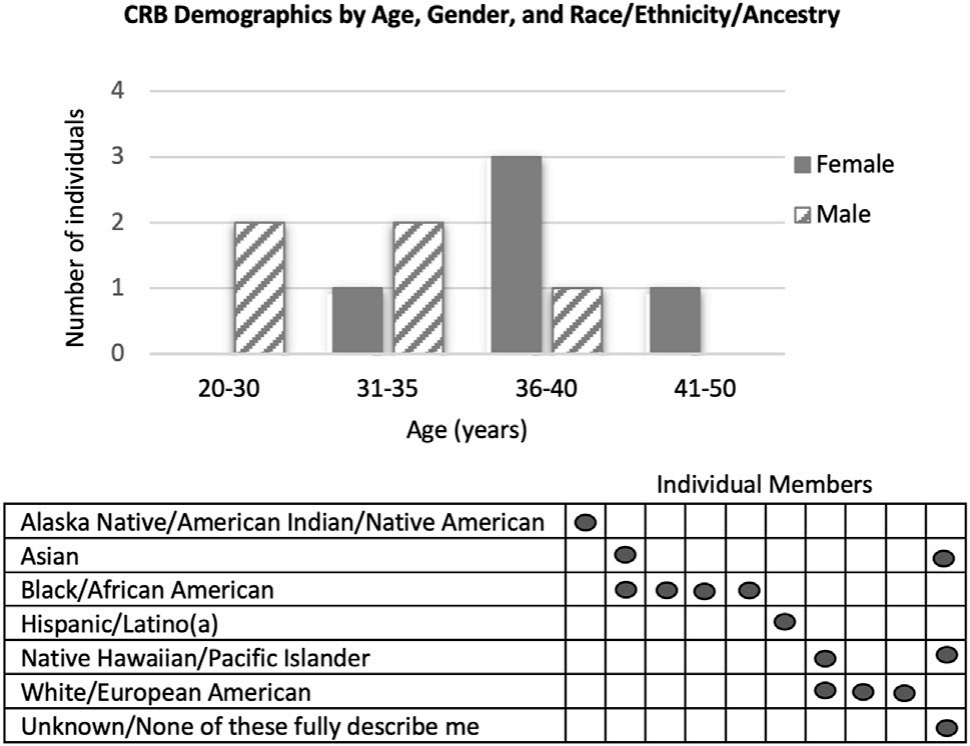
Demographics of CRB members by Age, Gender, and Race/Ethnicity/Ancestry. In the Race/Ethnicity/Ancestry table, each vertical column represents an individual member of the CRB.

Recruitment for a socio-demographically diverse CRB began in May 2020. Consultation with the Community and Stakeholder Engagement (CaSE) team at the North Carolina Translational and Clinical Sciences Institute (NC TraCS) at UNC-CH helped to optimize the design and reading-level of the recruitment materials. The CRB members were recruited over approximately six months from the Children’s Research Institute at UNC, a local church, online parent groups (Facebook and Reddit), and regional message boards (Reddit). Interested members were asked to complete a survey designed to invite members who could represent diverse communities and perspectives. CRB members (5M/5F; avg. 33.8 years see Figure 1) are parents (15 children; 0–16 years), represent urban, suburban, and rural communities, have diverse racial/ethnic backgrounds, varying health insurance coverage, a high school education or above, and views that ranged from “strongly supporting” to “not supporting” genomic screening of children as reported on the interest survey.

Based on review of interest survey responses, our recruitment methods were biased for individuals with positive or neutral attitudes towards genomic screening in childhood. While targeted messaging and snowball recruitment methods enabled successful recruitment of many diverse characteristics, we were only able to recruit one member who self-identified as “not supporting” genomic screening. Therefore, we continue to seek members with more critical views. Challenges related to COVID-19 were addressed via exclusively virtual participation.

### Formation and Relationship-Building

Initially, meetings focused heavily on building trust and familiarity, and creating a sense of community through a group resume activity that encouraged the team to recognize and share their knowledge, experiences, and motivations with the group. UNC investigators acknowledged historic neglect and abuse of racial and ethnic minorities in genetic and genomic science and shared their ongoing commitment to promoting diversity and inclusion in genomic research. The CRB and UNC team discussed their individual and shared goals, expectations, and timeline. Broad thought formation questions prompted the CRB to share their initial opinions of augmenting NBS with genomic sequencing. These included general excitement about potential benefits, as well as concerns about impact on insurance and the need for informed consent if sequencing of newborns became routine. As valued members of the research team who contribute invaluable insight, lived experience, and expertise, they are compensated at a rate of $50/hr.

### Capacity Building and Initial Activities

**FIGURE 2:**
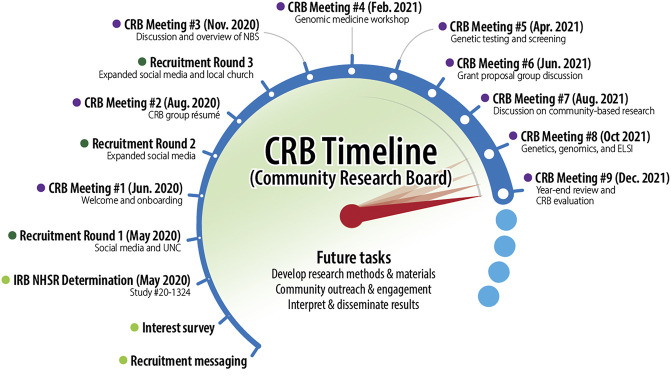
Timeline of CRB establishment and activities in 2020 and 2021.

After an initial formative period, the CRB met every other month in 2021 in the evenings *via* Zoom (see Figure 2). To facilitate bidirectional capacity building, the UNC-based AGBS investigators led a series of presentations to provide relevant background information for the CRB members. Topics included: newborn screening, genomic medicine and screening, ELSI, community-based participation, and academic research grant proposal development. Each topical presentation was followed by group discussion of key themes and questions. This enabled the CRB and UNC members to develop a mutual foundation of terms and concepts as well as issues of importance and concern for CRB members. Meetings were recorded and transcribed for later analysis. They were also summarized in a bimonthly newsletter that also included relevant news and information from the UNC team to maintain engagement between meetings.

Group discussions in 2021 focused on sharing knowledge and perspectives about a research proposal to develop a clinical pilot implementation of genetic screening for a healthy pediatric population. A research study with this aim and scope will require working closely with stakeholders, including parents, guardians, and caretakers, on many aspects of study design and development. We also discussed how the CRB would help to design accessible research tools and measures (e.g., interview guides and surveys) for mixed methods research to explore parental preferences for: 1) which conditions to screen for; 2) when and where screening should be done; 3) what and how results should be returned; and 4) educational strategies to facilitate the process of informed decision-making and parental consent.

In meetings over the course of 18 months, the CRB has shared their perspectives about thorny and contentious issues related to genomic sequencing of children. CRB members responded to discussion questions in the context of being offered screening for childhood-onset, medically actionable conditions for a healthy newborn. These early insights, shared below, will inform our ongoing research in this area including methods to elicit perspectives from broader stakeholder groups.

### Perspectives on Select Topics

#### Opt-In Versus Opt-Out

CRB members expressed frustration about the lack of information about NBS and agreed that transparency about issues such as false positives and false negatives, and privacy and data security, could improve their confidence about participating in expanded NBS.

“There are so many decisions made for people … without really consulting them … and there are so many people who do not recall being given any information … couldn’t there be a pamphlet or something at the doctor’s office?”

“I think the false positives prospect is why the follow ups need to be easily accessible. It is still stressful but easy to get a definitive answer.”

Other parents said they would rely on their doctors to help them make informed decisions.

“My gut reaction is yes, I’d like to pick the conditions, but honestly, not knowing exactly what conditions are being researched, and knowing that I may not know what 10 of those conditions even are, I think testing for as many as possible is best.”

A range of answers from the group illustrates a need to better understand the issues to choose effective and appropriate strategies for educating parents and facilitating informed decision-making.

#### Community Engagement

CRB members felt strongly that accessible alternatives (community-based and group offerings) to pediatric and family medicine clinics were needed.

“Working in the school system a lot of the families that I work with just don’t have the capacity to do anything extra … partnering with community agencies that have groups of people that already feel comfortable with one another could … reach a wide group of people that might typically not come for these kinds of information sessions.”

“Maybe something worth considering … is possibly illustrating these analogies and explaining these points through comics or something that the general public is not afraid of."

Community-based strategies used in other contexts (e.g., mobile vaccination buses) have clinical limitations for genomic screening, but the point was well made that creative engagement strategies are imperative for broad accessibility.

Insurance coverage for the cost of the screening test and other downstream costs also concerned the CRB members, both as parents and community representatives.

“I always go back to cost … to the patient [and] what’s covered by insurance.”

#### Privacy and Data Security

CRB members expressed trust in doctors and researchers and were open to providing their child’s de-identified DNA for research with a well-explained reason, though some noted they would need to be assured that their child’s data would not be misused.

“I’m uncomfortable with giving my child’s genetic info/DNA without having some sort of assurance that it will only be used for the sequencing and possibly anonymous data research.”

Members noted more concerns about providing DNA samples to companies and the government. One member identified perceived lack of transparency as a potential reason for declining to participate.

#### Which Conditions to Screen for and How to Deliver the Genetic Information?

In the context of early onset, medically actionable conditions, some CRB members were very concerned about severe conditions.

“I would want to know all of it. In the case of a package, I would want to know which ones create more of a strain on lifestyle. The name of the game is severity.”

Others were more concerned about having flexible options.

“I think it makes sense to have as many options as possible, so what works for one person might not work for another…”

## Discussion and Future Directions

Engaged Scholarship seeks to achieve health equity through shared decision making with stakeholder members of communities about research that is likely to impact the groups they represent (Goodman and Sanders Thompson, 2017). Engaging the CRB early in the research cycle has benefited all members. Parents have reported that their participation has given them a stronger sense of ownership of and advocacy in their own health care decision making. Parents and researchers report that the formative sessions contributed to a deeper trust and a sense of community and purpose. The research study benefits from an insightful model for education and outreach strategies that can be extrapolated to a broader population and a foundation from which to develop accessible and appropriate research tools and measures to address the significant variability in parental preferences, values, and beliefs about expanding NBS with genomic sequencing.

Parental engagement will be critically important to democratize access to expanded NBS. There is relevant concern that worsening health disparities contradict the principle that public health interventions should serve as equalizers. (Borry et al., 2009; Tarini and Goldenberg, 2012; Lewis et al., 2016; Evans et al., 2019; Moultrie et al., 2020; Peinado et al., 2020; Miller et al., 2021). Routine well-child interventions such as vaccinations and periodic screening for hearing, vision, and environmental exposures can have a profound effect on preventing individual morbidity and mortality and are also widely accepted because of their public health impact. Pediatric genomic screening has the potential to be adopted in a similar fashion if feedback from diverse parent stakeholders is sought and incorporated into the research process.

Willingness to participate in research is frequently shaped by cultural beliefs and personal and group experiences with health systems and research. CRB members are strategically positioned to build bridges between their communities and researchers, simultaneously increasing awareness of community perspectives and the benefits of participating in genomic research. Looking toward the future, we believe that engaging parents as partners throughout the genomic screening research process will reduce barriers to the uptake of highly actionable genetic information with the best chance of societal benefit.

## Data Availability

The original contributions presented in the study are included in the article/supplementary material, further inquiries can be directed to the corresponding author.
